# Biomimetic Exosomes: A New Generation of Drug Delivery System

**DOI:** 10.3389/fbioe.2022.865682

**Published:** 2022-05-23

**Authors:** Xudong Wang, Xian Zhao, Youxiu Zhong, Jiuheng Shen, Wenlin An

**Affiliations:** National Vaccine & Serum Institute (NVSI), China National Biotech Group (CNBG), Beijing, China

**Keywords:** exosomes, drug delivery system, biomimetic exosomes, smart, large scale production

## Abstract

Most of the naked drugs, including small molecules, inorganic agents, and biomacromolecule agents, cannot be used directly for disease treatment because of their poor stability and undesirable pharmacokinetic behavior. Their shortcomings might seriously affect the exertion of their therapeutic effects. Recently, a variety of exogenous and endogenous nanomaterials have been developed as carriers for drug delivery. Among them, exosomes have attracted great attention due to their excellent biocompatibility, low immunogenicity, low toxicity, and ability to overcome biological barriers. However, exosomes used as drug delivery carriers have significant challenges, such as low yields, complex contents, and poor homogeneity, which limit their application. Engineered exosomes or biomimetic exosomes have been fabricated through a variety of approaches to tackle these drawbacks. We summarized recent advances in biomimetic exosomes over the past decades and addressed the opportunities and challenges of the next-generation drug delivery system.

## 1 Introduction

During the past several decades, naked therapeutic agents, including small molecule drugs and biomacromolecule drugs, were used to prevent, diagnose, or treat different diseases. However, some limitations such as low solubility, poor stability, short circulating half-life, and poor biodistribution of these drugs contributed to limited efficiency (Zhao et al., 2021). In order to solve the aforementioned problems, a wide variety of delivery systems have been constructed in recent years to enhance the therapeutic effect and reduce side effects (Wang et al., 2021; Yi et al., 2022). Among them, nanotechnology was introduced into medicine and, therefore, improved treatment. Various synthetic nanoparticles, such as liposomes (Sur et al., 2014; Dammes et al., 2021), micelles (Gong et al., 2012; Kataoka et al., 2012), and self-assembled peptides (Cully, 2015; Worthington et al., 2017), and inorganic materials, such as graphene (Xiaoming et al., 2008; Panwar et al., 2019) and graphene-like 2D materials (Huang et al., 2021), are used to carry different cargo (Kuang et al., 2020; Zhou et al., 2021). Over the past few decades, nanocarriers for drug delivery have found applications in clinical use (Wicki et al., 2015; Min et al., 2015). The approved nano-drugs Doxil, Lipusu, and Abraxane mainly utilized liposomes as carriers. With the outbreak of COVID-19, liposome-based mRNA vaccines (Flemming, 2021; Gebre et al., 2021) were applied against COVID-19 (Flemming, 2021; Gebre et al., 2021); liposomes have drawn wide concern. However, these exogenous nanomaterials encountered several biological barriers and were quickly cleared from the blood upon delivering drugs to targeted locations (Peng et al., 2013). Studies have shown that less than 1% of nanomedicine reaches the target tissue (Huang et al., 2020). The failure of clinical trials of synthetic nanomedicine is mainly due to the difference in the biological barrier and immune system of humans and animals (Foulkes et al., 2020). By coupling with PEG to nanoparticles to prolong the circulation time of nanomedicine in the blood (Tam et al., 2013; Suk et al., 2016), antibodies (Sun et al., 2014), peptides (David, 2017), and aptamers (Subjakova et al., 2021) were modified at the surface of nanocarriers to increase the targeting ability and improve biodistribution. Nevertheless, the delivery efficiency of modified nanoparticles to targeted locations was still limited. This was because repeated injections of PEGylated nanomedicine caused faster blood clearance and a significant increase in the amount of aggregation in the liver and spleen (Ishihara et al., 2009). The long cycle capability of nanomedicine was therefore dramatically decreased, which was endowed by introduction of PEG onto nanoparticles. In addition, serious toxic and side effects in the body were caused for the encapsulated drug on account of the change of its pharmacokinetic behavior.

In order to tackle these issues, numerous biological carriers have recently been applied to achieve intracellular delivery of nanomedicine. Biological sources of carriers have received widespread attention due to their ability to alter biological distribution, uptake, and cause controllable responses (Bu et al., 2019). These vectors included membrane fragments (Park et al., 2021), exosomes (Liang et al., 2021), and viruses (Sokullu et al., 2019), while further applications of these virus were restricted due to strong immunogenicity (Bulcha et al., 2021). Exosomes were one kind of vesicles which were secreted from the cell membrane by cells with a particle size in the range of 40–160 nanometers. Since vesicles were derived from cells, exosomes have similar structures and properties to those of cells (Antimisiaris et al., 2018). Exosomes contained phospholipid bilayers and cytoplasmic proteins, membrane proteins, RNA, DNA, and other components similar to parent cells, but exosomes have no organelles. Exosomes played key roles in many biological processes under a variety of physiological and pathological conditions, and were considered to be a new mechanism for cell-to-cell communication (EL Andaloussi et al., 2013). Exogenous materials, including proteins, lipids, and genetic materials, could be delivered into recipient cells through exosomes. As a consequence, exosomes can be used as biomarkers for disease diagnosis (Ge et al., 2020) and prognosis (De Rubis et al., 2019). In addition, the large capacity and efficient ability of exosomes to exchange protein genes with cells give them the potential to be used clinically as a carrier for gene and drug delivery (Jie et al., 2021). Exosomes showed higher biocompatibility and lower toxicity in comparison to synthetic vehicles (Li et al., 2019). The surface of the exosome membrane contains all sorts of proteins that interact with integrins, allowing exosomes to overcome various biological barriers in the body, including clear monocytes, cell adhesion, permeation of tissues, spreading into the blood, and even passing through the blood–brain barrier. Thus, exosomes were regarded as natural vehicles to transport not only RNA but also DNA toward cells.

Although exosomes show great potential in treatment, their targeting capability of natural exosomes was relatively low, limiting further clinical applications (Liang et al., 2021). Over the years, a series of engineered exosomes have been developed to increase drug loading rates and improve the ability to target sites (Sterzenbach et al., 2017). By means of chemical modification, genetic engineering, and physical method, the exosomes were endowed with more functions and improved the targeting efficiency.

There were some major drawbacks of exosomes before clinical applications. For exosomes, it was virtually impossible for the preparation of vesicles with the exact duplicate for biomedical applications owing to the complex structures (De La Peña et al., 2009). In addition, the exact working mechanism was hard to elucidate due to its intricately pathophysiological functions. As a consequence, their development of extracellular vesicles may be hampered by their short- and long-term side effects caused by the unknown components for clinical practice. Furthermore, no standard methodologies existed to produce pure exosomes which met clinical applications to date. On account of different methods of preparation, the characteristics and functions of generated extracellular vesicles were also alike (Yong-Jiang Li et al., 2021). Thus, there is an urgent need to develop a common strategy to fabricate exosomes by researchers in the near future. Furthermore, extracellular vesicles which originated from organisms comprised of a mixture of a variety of extracellular vesicles derived from cell types of sorts, causing difficulty for the isolation of a single vesicle population to determine the dominant inducer of a particular function. Furthermore, they were not sufficient to generate at a large scale because of the low production yield in naturally secreted extracellular vesicles, which limited their further application (Antimisiaris et al., 2018).

**SCHEME 1:**
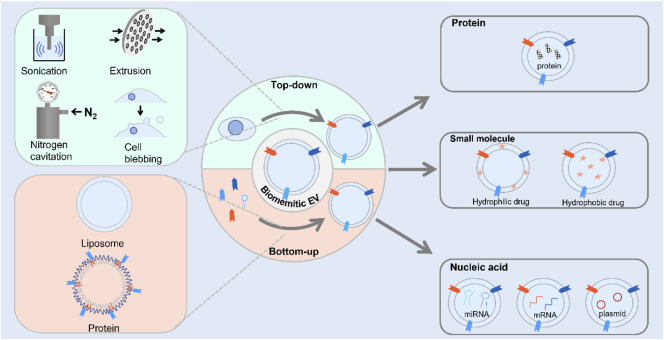
Scheme illustration of the route for the preparation of biomimetic exosomes and its application to deliver therapeutic agents.

In an attempt to overcome the shortcoming of exosomes which acted as cargo, artificial exosomes have been developed as vehicles to different delivery agents. In this review, the progress of biomimetic exosomes for drug delivery over the past decades was reviewed (Scheme 1). First, we introduced the preparation methods of biomimetic exosomes including chemical methods and genetic engineering methods. Second, we introduced the application scenarios of biomimetic exosomes. Finally, we summarized the advantages and challenges of biomimetic exosomes, aiming to provide inspiration in developing delivery systems for cell-free therapy.

## 2 Preparation of Biomimetic Exosomes

### 2.1 Strategies for Biomimetic Exosome Preparation

Compared to exosomes, biomimetic exosomes with high-titer production were used to import therapeutic agents into cells. With the rapid development of nanotechnology, a great deal of research efforts has been made to develop strategies of preparation methods for constructing novel functional materials. For the fabrication of materials at nanoscale, the materials were prepared with minimum dimensions with particle size ranging from 1 to 100 nm. A series of approaches have been used to fabricate nanoscale biomimetic exosomes with different functions from cells and small molecules. Currently, the method for artificial exosome preparation could be divided into three categories: top-down, bottom-up, and biological strategies.

#### 2.1.1 Top-Down Approach

The top-down approach for the preparation of materials at nanometer scale was that larger materials were broken into nanoscale structures. The exosome was secreted by various kinds of cells, with similar components to cells such as proteins and lipids. Therefore, cells were usually used as bulk materials for the preparation of artificial exosome *via* the aforementioned strategy. Cell membrane sheets were obtained by breaking cells to form vesicles. The composition of the fabricated nanovesicle membrane showed high similarity with natural exosomes because both of them were produced from cells. The generated artificial exosomes contained proteins, nucleic acids, and lipids. These artificial exosomes possessed the same biological complexity of exosome, whereas little heterogeneity was observed. On the basis of this approach, several strategies have been used to produce nanovesicles, including extrusion, filtration, microfluidic device, sonication nitrogen cavitation, and chemical-induced cell blebbing.

##### 2.1.1.1 Extrusion-Based Strategies

Filter extrusion (using polycarbonate membrane filters) was extensively applied with dimension reduction. This method was ready to operate and produced nanomaterials with controllable size. For the preparation of nanovesicles, cells at the micron level were extruded by polycarbonate membranes of defined pore size with sequential extrusion, obtaining homogeneous nanovesicles with nanoscale size. Generally, a commercial liposome extruder was used to accomplish the extrusion process. In addition, several devices have been used for preparing artificial exosomes at large scale.

**FIGURE 1:**
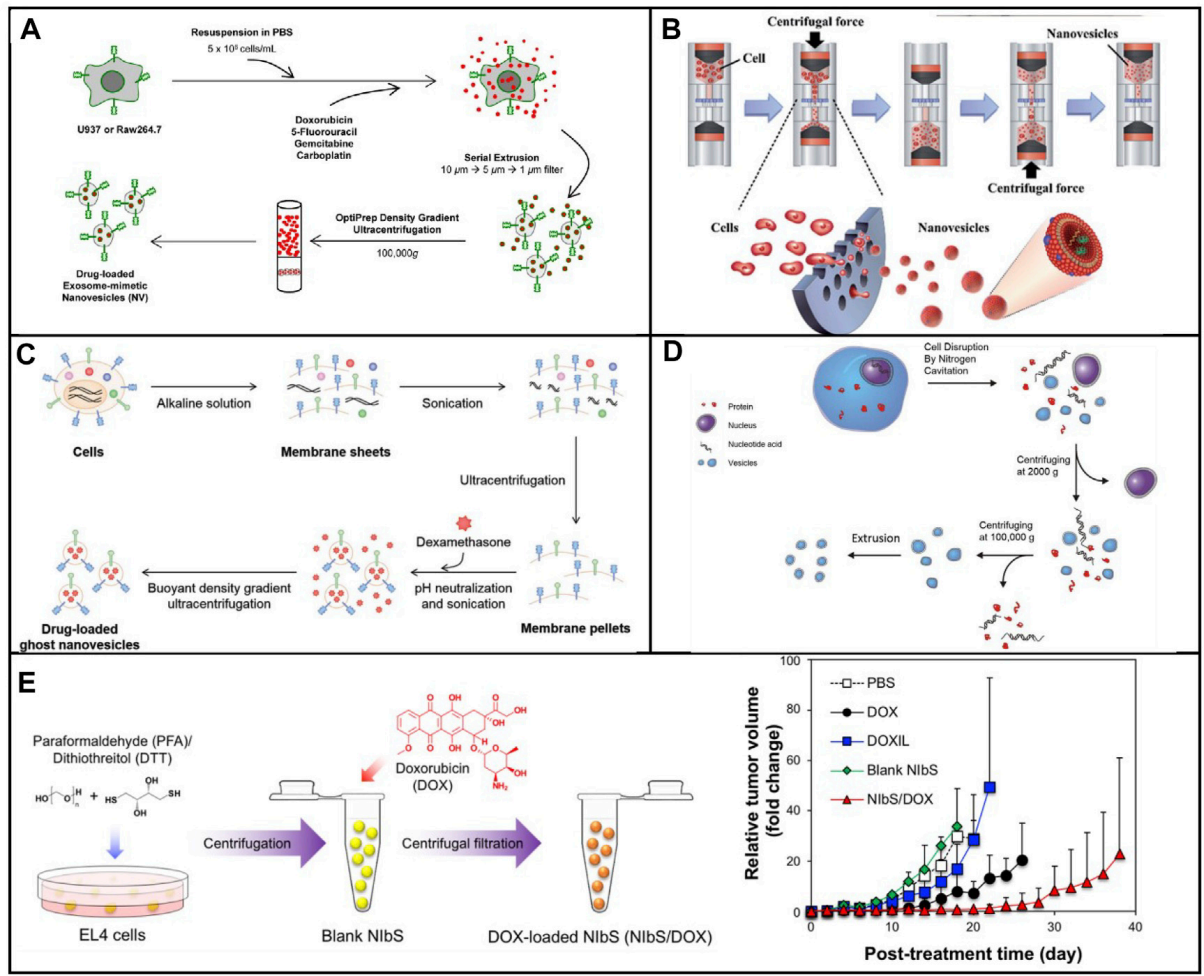
**(A)** Schematic illustration of the procedure for the generation of NV and chemotherapeutics-loaded NV (Jang et al., 2014). **(B)** Schematic process of nanovesicle generation (Jo et al., 2014). **(C)** Schematic illustration of the procedure for generation of ghost nanovesicles (ghost NVs) and dexamethasone-loaded ghost NVs (Go et al., 2019). **(D)** Schematic shows a process to generate a uniform size of nanovesicles including cell disruption, differential centrifugation, and extrusion (Gao et al., 2016). **(E)** Production and drug loading of NIbS derived from cancer cells and efficient therapeutic efficacy against murine EL4 tumors by EL4-derived NIbS/DOX (Ingato et al., 2018).

In 2014, nanosized vesicles loaded with chemotherapeutic drugs were developed by the GHO Group utilizing sequential extrusion through polycarbonate membrane filters with a decrease in pore sizes, starting at monocytes or macrophages in the presence of doxorubicin (Figure 1A) (Jang et al., 2014). As compared with the yield of natural exosomes from an identical number of cells, the bioinspired nanovesicle yield was increased 100-fold. The obtained nanovesicles ranged in size from 120–130 nm, and exosome marker proteins such as moesine, CD63, and TSG101 also existed in nanovesicles. These nanovesicles were similar to natural exosomes in particle size, morphology, and protein contents. Owing to endothelial CAMs as raw materials, the generated nanovesicles which maintained the topology of plasma membrane protein showed the targeting ability toward tumor site. By means of the obtained nanovesicles, chemotherapeutic drugs could be efficiently delivered to the site of the tumor with less adverse effects. Importantly, the curative effect of nanosystems reduced obviously as plasma membrane proteins were removed. Importantly, the plasma membrane proteins of the nanovesicles were removed by trypsin to reduce their therapeutic effects**.** Taken together, the bioengineered nanovesicles as novel exosome-mimetics were successfully transferred chemo drugs toward malignant tumors.

Although extruding cell *via* microchannel was successfully employed to generate nanovesicles, the critical challenge for clinical applications was both the delivery efficiency and the scale-up production. The fabrication processes should be well controlled in large-scale nanovesicle preparation with uniform quality. Recently, the Park group developed a method to prepare a massive range of artificial exosome with culture cells through utilizing a common centrifuge (Figure 1B) (Jo et al., 2014). Compared with hand extruder, the extrusion force by centrifugation could be controllable to extrude cells. Once the centrifuge worked, cells were directly broken into nanovesicles by hydrophilic micro-size pores. Two much of the intracellular contents in the generated nanovesicles were observed when compared with natural exosomes. In addition, the nanovesicles could be selected to deliver their contents, including Oct3/4 and Nanog, to recipient cells and used for activation of the signaling pathway. A huge amount of nanovesicles, which were produced by using a centrifuge and polycarbonate structure with the filter, could be utilized for practical applications including drug delivery. The source cell which expressed specific targeting molecules endowed the produced nanovesicles with a targeting capability.

In addition to cells being raw materials, the lysed cell membrane could be used to form nanovesicles, which has the capability to replicate the functionalities of cell. With the development of the cell membrane coating technology, nanovesicles were prepared by coating various types of cell membranes onto nanoparticles. The generated nanovesicles have been demonstrated to efficiently deliver the drug because of their abilities to prolong circulation time. In addition, proteins on the cells endow that biomimetic nanovesicles could interact with a series of disease substrates, leading to the home capability toward disease. Additional functions were provided by nanoparticles in the nanovesicles to enhance their effect in nanomedicine. A series of nanovesicles based on cell membrane–coated nanoparticles have been constructed to treat multiple diseases (Rao et al., 2017; Rao et al., 2018; Ai et al., 2021; Meng et al., 2021). For example, Rao et al. (2020a) developed hybrid cell membrane nanovesicles from cancer cells and macrophages to restrain tumor recurrence and metastasis after surgery. Cancer cell membranes with high-affinity SIRPα variant overexpression, platelet, and M1 macrophage–derived nanovesicles were sonicated and extruded through 100-nm pores. SαV-C-NVs, M1-NVs, and P-NVs have endowed the hybrid nanovesicles with long systemic circulation time, specific targeting, and activation to combat cancer. The hybrid nanovesicle could act as a platform to deliver bioactive molecules. In addition, a biomimetic exosome as nanodecoy for COVID-19 was produced by the extrusion of both 293 T-cell membranes with overexpressed ACE2 and human monocytes (Rao et al., 2020b). ACE2 on the surface of biomimetic exosomes was capable of hijacking the S protein–mediated viral infection. Meanwhile, biomimetic exosomes showed that the neutralization capability toward inflammatory cytokines significantly suppressed immune disorder and lung injury against COVID-19.

##### 2.1.1.2 Sonication-Based Strategies

Sonication was a widely spread method that used a strong bath sonicator or ultrasonic probe to reduce particle size to yield nanosized particles. Sonication was another common approach for making liposome. Cells could be broken into cellular fragments in the preparation of artificial exosomes at the beginning. Then, the cell fragments were subjected to sonication to assemble into nanosized vesicles. In 2019, Go et al. (2019) constructed a novel approach to synthesize artificial exosomes by removing the unwanted cellular content. First, the cells were treated with alkaline solution, fragmented, and disassembled into membrane sheets (Figure 1C). Meanwhile, cellular contents including cytoplasm protein, nucleoprotein, and nucleic acids were completely released into an aqueous solution. Second, the cell membrane fragments in aqueous media were harvested by ultracentrifugation. Finally, membrane sheets were subjected to sonication with or without the addition of dexamethasone. These vesicles showed high efficiency in the treatment and production yield. In addition, undesired luminal cargos were separated, resulting in the reduction of potential adverse effects and facilitating drug loading. Moreover, artificial exosomes could be generated from various types of cells loading other drugs for the treatment of other diseases, expanding its practical application in clinical environment.

##### 2.1.1.3 Nitrogen Cavitation-Based Strategies

Membrane proteins play significant roles in selectively delivering therapeutic agents into the site of lesion. A series of membrane proteins was involved in signal pathways to trigger the pathway activation or inhibition. The activity of membrane proteins should be retained in the process of generating nanovesicles. The nitrogen cavitation method was an effective approach for the disruption of cells and maintained the biological function of membrane proteins. Nitrogen cavitation referred that nitrogen could be dissolved in the cytoplasm of the cells under high pressure. Subsequently, nitrogen bubbles were formed in the cytoplasm cell suspension, which was abruptly exposed to atmospheric pressure such that nitrogen bubbles were formed in the cytoplasm, leading to fragmentation of cells. Gao et al. (2016) proposed a general approach to prepare cell-derived vesicles by employing nitrogen cavitation (Figure 1D). To generate nanovesicles with the capability of reducing acute lung inflammation and injury, active neutrophils were used as source cells. In comparison with extracellular vesicles, 100-fold was achieved in the production yield of nanosized vesicles loaded with dexamethasone. This proposed strategy showed great potential for the development of personalized nanomedicine to treat the given disease.

##### 2.1.1.4 Cellular Blebbing–Based Strategies

Cellular blebbing was a large and spherical outgrowth of cell membrane, which occurred throughout the lifecycle of different cell types. Cell blebbing was observed in the presence of chemical or multiple mechanical stimulations. Although giant plasma membrane vesicles had been used, a wide size range caused a barrier for the practical application. To address the aforementioned drawback, the chemical stimulation method was used by Ingato et al. (2018) to efficiently produce nanovesicles induced by sulfhydryl-blocking (NIbS) (Figure 1E). Intracellular and extracellular osmotic pressure was used to control the blebbing process. The resulting nanovesicles could deliver chemotherapeutic agents to the tumor, improving cellular uptake and facilitating drug release in the cells. Furthermore, the accumulation in major organs was avoided by employing the nanosystem as compared to commercial liposomal formulation. The drug-loaded nanosystem obviously reduced the growth rate of tumor, resulting in the increase of the survival of tumor-bearing mice. Nanosized chemical-induced vesicles could be considered for mass production of nanovesicles with efficient, quick, and simple harvest and purification. In addition, NIbS were utilized in various therapeutic scenarios such as immunotherapy, gene therapy, and cell therapy.

#### 2.1.2 Bottom-Up Approach

To date, the bottom-up approach was that small molecules were dissolved in an organic solvent and then precipitated on addition of an anti-solvent in the presence of a stabilizer. There are a variety of strategies of this approach including liposome conjugated with specific peptide, liposome coupled with antibody, NPs embedded with specific protein, liposomes modified with membrane proteins, and fully synthetic nanovesicles.

##### 2.1.2.1 Liposomes Coupled With Peptides

**FIGURE 2:**
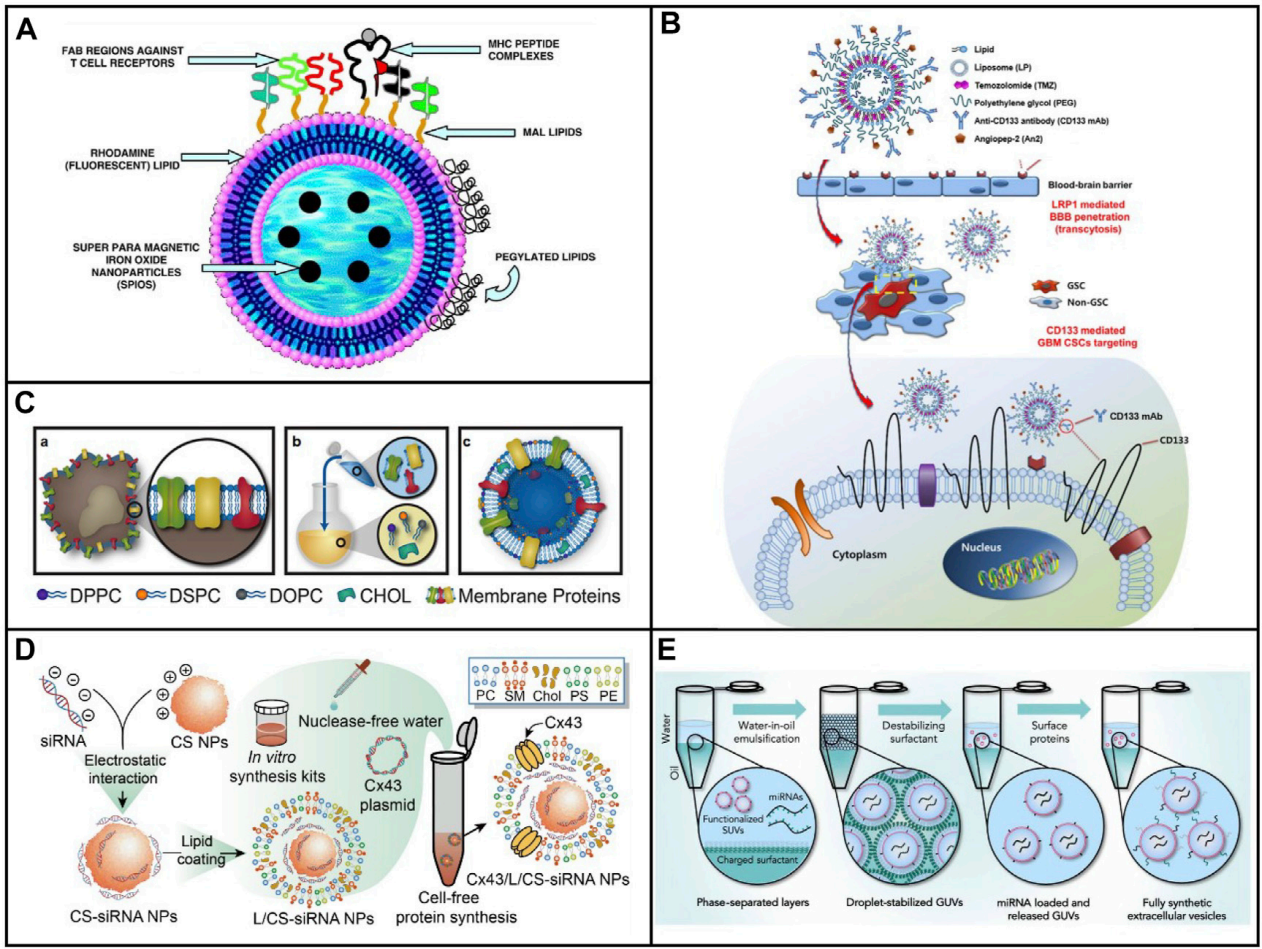
**(A)** Schematic of immuno-magnetoliposomes as artificial exosomes. **(B)** Scheme of a dual-targeting immunoliposome encapsulating TMZ and mechanism for the delivery of TMZ to GSCs in dual-targeting immunoliposome formulation (Moreira et al., 2002). **(C)** Scheme of leukosome synthesis and formulation (Molinaro et al., 2016). **(D)** Schematic illustration of the preparation procedure for the construction of exosome mimetic Cx43/L/CS-siRNA NPs (Lu et al., 2019). **(E)** Schematic illustration of the fsEV formation process inside w/o droplet compartments produced by mechanical emulsification (Staufer et al., 2021).

Peptides could perform a lot of functions such as the targeting capability, the ability to penetrate cellular membrane, the stimuli responsive ability, and the therapeutic ability because of these specific sequences. Thus, the function of exosomes could be mimicked by liposomes after modification by peptides with specific sequences. It was an easy way to construct biomimetic exosomes with various functions owing to its highly modular design. Liposomes modified with MHC Class I/peptide complexes served as biomimetic exosomes to activate and expand functional antigen-specific T cells at sufficient cells (De La Peña et al., 2009). As shown in Figure 2A, the peptide was conjugated on the maleimide group in the liposome and sulfhydryl group, which was introduced to peptides with Tarut’s reagent. Interestingly, the sulfhydryl group was introduced *via* the reaction between Tarut’s reagent and primary amines without altering charge characteristics. The fabricated biomimetic exosomes have a similar size as that of exosomes as well as similar functions for activation and expansion toward T cells. Moreover, the fluorescent and magnetic elements were introduced into biomimetic exosomes, which endow the targeting and traceable capability. In addition to covalent modification of liposome with peptides, peptides could be inserted into liposomes at 60°C. Moreira et al., 2002 inserted a peptide (antagonist G) into a liposome through the postinsertion technique. Compared with antagonist G-liposome using the conventional coupled technique, the generated antagonist G-liposome exhibited high binding under the same number of peptides, resulting in higher cytotoxicity toward H69 cell line.

##### 2.1.2.2 Liposomes Coupled With Antibody

In addition to peptides, antibodies and proteins were composed of a variety of amine acids, which could also be covalently modified through different groups. As a consequence, both antibodies and proteins could be modified onto the surface of liposome *via* coupling and the postinsertion technique. Kim et al. (2018) developed angiopep-2 (An2) and anti-CD133 monoclonal antibody (CD133 mAb) conjugated liposomes, which could be used to deliver temozolomide for the treatment of glioblastoma multiforme (Figure 2B) (Moreira et al., 2002). An2 showed high targeting to low-density lipoprotein receptor-related protein on the blood–brain barrier (BBB), while anti-CD133 was capable of targeting toward CD133, which was highly expressed on the cancer stem cells of glioblastoma multiforme. Dual targeting liposomes endowed them to deliver drugs across BBB and reach the cancer stem cells. Much better therapeutic efficacy and fewer side effects were observed after treatment with dual targeting liposome. The system provided a convenient approach to fabricate biomimetic exosomes with dual function.

##### 2.1.2.3 Liposome Embedded With Membrane Proteins

The bottom-up strategy has offered a novel solution to mimic the function of natural nanoparticles. Nevertheless, the complexity of membranes which was on the surface of liposomes was still not reproduced. In order to mimic the function of natural NPs, the complex synthetic route was required to add multiple elements onto the surface of liposome. Although the top-down strategy has provided a simple way to endow NPs with several bioactive functions, several limitations still existed by utilizing these approaches in the control of physical parameters, various kinds of cargo loading, and standardized preparation and storage approaches. In view of this, Molinaro et al. (2016) developed a biomimetic vesicle for the first time which incorporated membrane proteins into a lipid bilayer. As can be seen in Figure 2C, the nanovesicle could be fabricated by using the thin layer evaporation approach and integrating proteins derived from the leukocytes’ plasmalemma into a synthetic phospholipid bilayer. Not only primary but also immortalized immune cells were used to extract the membrane proteins, which were utilized onto the surface of biomimetic vesicle. Subsequently, a mixture of cholesterol, synthetic choline-based phospholipids, and purified membrane proteins was assembled through thin layer evaporation methods. The generated nanovesicles called leukosomes showed the well-targeting property of inflamed endothelia. The proposed route offered a universally synthetic method to produce biomimetic exosomes, which maintained the multiple functions of natural membranes.

##### 2.1.2.4 Liposome-Coated Polysaccharides

Polysaccharides are biological polymers which consist of a lot of small monosaccharides. Polysaccharides include glycogen, inulin, hyaluronic acid, heparin, chondroitin 4 sulfate, gamma globulin, chitosan, alginate, dextran, cellulose, gelatin, and starch, and are widely used in biomedical applications. Chitosan was a positively charged polysaccharide which could bind polyanionic molecules, for example, nucleic acids. Liposome-based artificial exosomes could be used as cargo to carry drug or gene delivery. Lu et al. (2019) developed liposome-coated chitosan NPs to biomimetic exosomes for the delivery of siRNA. A plasmid that encoded, transcribed, translated, and integrated a transmembrane protein called connexin 43 (Cx43), transcription, translation, and integration was introduced into liposomes in the lipid layers in biomimetic exosomes (Figure 2D) (Lu et al., 2019). Integrated Cx43 worked functionally in cellular transport and facilitated the delivery of siRNA in EM to Cx43-expressing U87 MG cells. The EM exhibited high siRNA delivery efficiency and biocompatibility. Despite limited delivery efficiency than commercial transfection Lipo 2000 reagent, the strategy formulated EM using a cell-free protein synthetic approach and advanced the development of artificial exosomes as biomimetic nanocarriers.

Hyaluronic acid (HA) is a natural negatively charged hydrophilic polysaccharide with appreciable water solubility. Owing to the specific interaction of HA to CD44, a cell surface receptor, HA, could be served as a targeting moiety in nanomedicine for cancer therapy. Ravar et al. (2016) developed a HA-coated liposome to deliver paclitaxel through electrostatic interaction. The prepared biomimetic exosomes demonstrated high uptake efficiency in CD44 overexpressed cells, resulting in the improved cytotoxic activity compared to free drug and PTX liposomes without HA. Furthermore, HA-coated PTX liposomes showed high accumulation in the tumor area because of a high level of CD44 in tumors, and promising results on antitumors have been achieved in 4T1 tumor-bearing mice. Therefore, modifying liposomes with polysaccharides was an effective strategy to construct the biomimetic exosomes with specific functions.

##### 2.1.2.5 Protein Vesicles

Generally, vesicles were prepared from lipids (liposomes) or polymers (polymersomes). In order to construct membrane-bound compartments, proteins were considered ideal building blocks. A vesicle to mimic the properties of cells has been developed by Huang and his colleagues through self-assembly of conjugated protein-polymers. In this design, a protein was covalently linked with a polymer *via* chemical reaction between mercaptothiazoline-activated amide and primary amine groups. The vesicle was formed through self-assembly at a constant aqueous/oil interface. The vesicles possessed the properties including molecule encapsulation, semipermeable nature, and protein synthesis guided by gene and enzyme catalysis. However, the function of proteins has been lost after chemical modification (Huang et al., 2013). To retain the biological activity of proteins on the surface of vesicles, a novel method has been used by Dautel and Champion (2021). Two recombinant fusion proteins, elastin-like polypeptide and globule-Z_E_, were utilized to form vesicles. A glutamate-rich leucine zipper (Z_E_) that could bind arginine-rich leucine zipper (ZR) was genetically fused with globules. The hydrophilic globule-ZE domain and the hydrophobic domain, Z_R_-ELP, were connected *via* the leucine zipper pair to form an amphiphilic complex. Hydrophobic conformational changes of ELPs were induced upon heating, leading to lower critical solution temperature (LCST) behavior. During the heating from 4°C to room temperature, the vesicle occurred *via* a transient coacervate phase owing to the LCST behavior. The aforementioned approach showed advantages such as tunability *via* genetic engineering and the incorporation functional proteins with maintained activity.

##### 2.1.2.6 Fully Synthetic Exosomes

To precisely mimic the functions of natural exosomes, Staufer et al. (2021) constructed a complementary synthetic biology method to generate biomimetic exosomes with full function of natural exosomes (Figure 2E). The fully synthetic exosomes were composed of proteins, RNA, and lipids, which contained the most abundant lipid composition in natural exosomes. The ratio of various lipid components was cholesterol: SM: DOPC: DOPS: DOPE: DOPG: PA: DAG: DOPI = 43:16:15:11:6:5:2:1:1. Thereafter, lipids were assembled through the charge-mediated vesicle assembly technology (Figure 2E). Abundant encapsulation efficiencies were achieved for diverse kinds of biomolecules. Furthermore, it offers precise quantitative control over vesicle composition and, therefore, over the biochemical and biophysical phenotype of the vesicles. Lastly, the resulting synthetically assembled vesicles in many parts closely resemble naturally occurring lipid carriers such as EVs.

#### 2.1.3 Biological Approaches

##### 2.1.3.1 Assembly Mechanism of Virus

Viruses consist of nucleic acids and proteins, having a particle size in the range of 20–400 nm. A protein called capsid could package their nucleic acid including DNA or RNA with single strand or double strand, thus protecting them from damage by external factors. In some cases, the lipid bilayer membrane of host cells coats viruses such as HIV and influenza. During the formation of viruses, capsid was assembled, and subsequently nucleic acid genome was packaged into capsid. Finally, the cell membrane was coated on the surface of capsid if enveloped. Generally, a large number of viruses whose genomes were single strand assembled spontaneously around their capsid. Nucleic acids with double strands having high charge density prevented spontaneous encapsulation. Consequently, an empty protein capsid was formed in numerous dsDNA virus assemblies (procapsid), and thereafter, DNA was pumped into capsid with the help of a molecular motor.

Viruses could act as vehicles to deliver nucleic acids for gene therapy. Gene therapy utilized genetic materials to replace a defective gene for the treatment of genetic diseases. Significant progress has been achieved in the field of treatment of diseases, which was incurable previously. The common genetic materials included mRNA and plasmid DNA in gene therapy. Gene-delivery vectors were needed for gene therapy due to their easy degradation. Adeno-associated virus (AAV) and retroviruses were widely used to transfer genes across the cell membrane. Nevertheless, the biggest challenge *via* AAV as gene vector was the limited efficiency because AAV could be degraded or neutralized rapidly owing to host immune response. The delivery efficiency could be enhanced after the envelopment of AAV to protect them from the immune system. Recently, a virus could evade neutralizing antibodies after envelopment with cell membranes from host cells. Endosomal-sorting complexes required for transport (ESCRT) involved the process to envelop the virus. Interestingly, ESCRT was also involved in the formation of exosomes. Thus, exosomes produced from virus-infected cells contained virus-derived nucleic acids, viral proteins, and even viruses. During the process of viral invasion, exosomes played several roles such as the transportation of virus into target cells and changing the physiology of target cells to facilitate infection. There were two mechanisms for exosomes to promote viral infection. One was that exosomes could interact with the ligand on the surface of the cell membrane to elicit down signaling events, and another was to promote cell uptake and intracellular release through membrane fusion or receptor-mediated endocytosis. In addition, the reduction systemic toxicity was achieved by employing engineering exosome with targeting element to improve the targeting ability.

##### 2.1.3.2 Biomimetic Exosomes Derived From Virus

**FIGURE 3:**
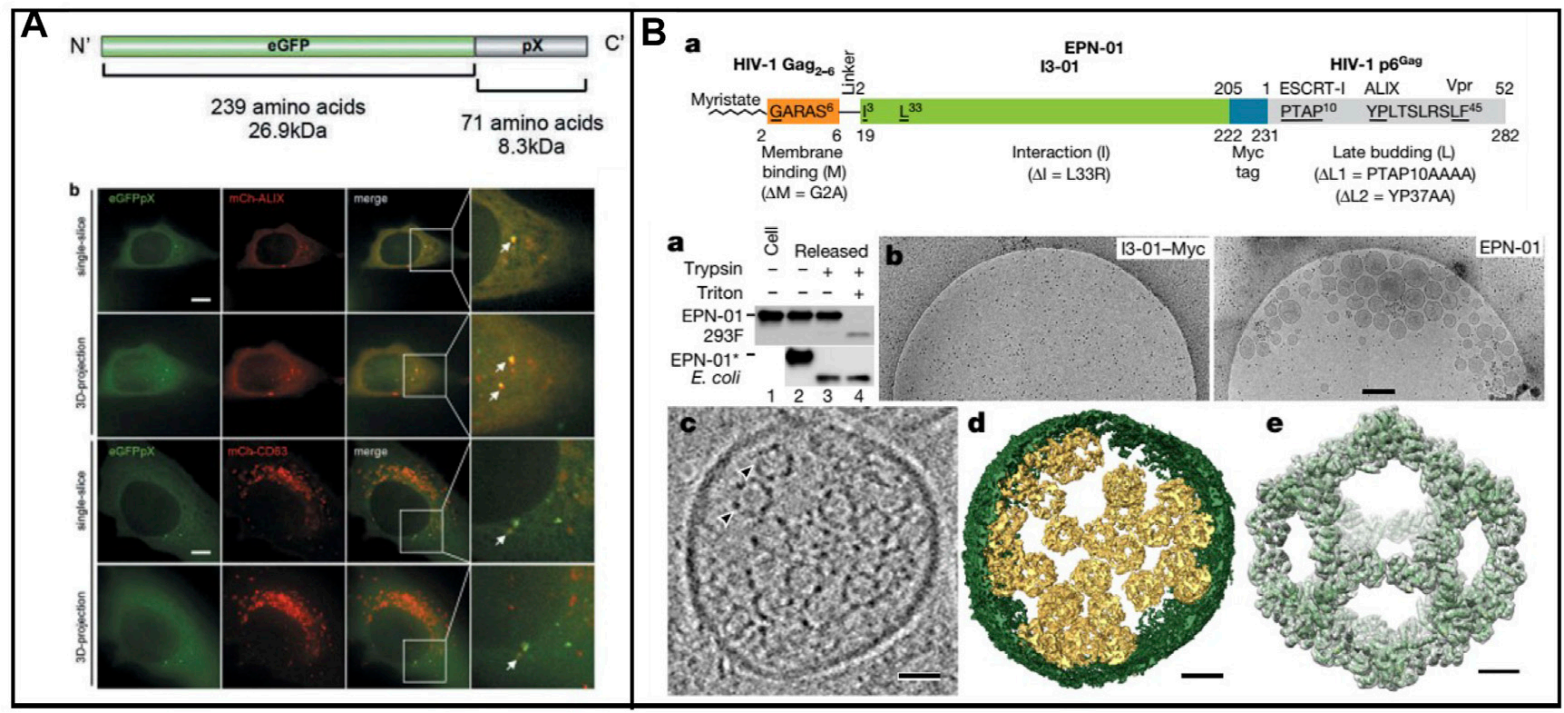
**(A)** Schematic representation of the enveloped nanocages and representative cryo-EM images showing exosome/EPNs (Jiang et al., 2020). **(B)** Scheme of the recombinant eGFPpX protein and eGFPpX co-localized with the ALIX and CD63 in live cells. mCh-ALIX and mCh-CD63 were stably expressed in Huh7-eGFP and Huh7-eGFPpX cells (Votteler et al., 2016).

A lot of enveloped RNA viruses were released from host cells through ESCRT, which displayed unique characteristics. In many biological events, including the biogenesis of multivesicular bodies (MVBs), cytokinesis, and retrovirus budding, ESCRT is essential (Vietri et al., 2020). Significantly, the host cell membrane could be hijacked by the nonenveloped RNA virus in nonlytic release. Recently, a classic nonenveloped virus (Hepatitis A virus, HAV) was reported its release in the form of quasi-enveloped HAV. Furthermore, the Long group found that the exosome markers such as CD9, CD81, and CD63 existed in an enveloped HAV particle (Figure 3A) (Jiang et al., 2020). ALIX, a protein associated with ESCRT, was also found in enveloped HAV, demonstrating that ESCRT involved the formation of enveloped virus. In addition, pX, which existed on the surface of the virus capsid, could direct the virus into exosome-like EVs. In the meantime, ALIX was involved in the aforementioned process. This study provides a fresh insight into large proteins packaged into vesicle-derived cells.

In addition, nonenveloped viruses were loaded into exosomes to form biomimetic exosomes; protein nanocages could be enveloped into exosome-like vesicles *via* ESCRT. Votteler et al. (2016) developed the design of self-assembling protein nanocages that direct their own release from human cells inside small vesicles in a manner that resembles some viruses (Figure 3B). Three distinct functions of protein sequence that served as membrane binding, self-assembly, and recruitment of ESCRT were required in the biogenesis enveloped protein nanocages (EPNs). Fused peptide sequences (EPN-01) with the ability of membrane binding and ESCRT were constructed. To promote membrane binding, N-myristoylation signal corresponding to the first six amino acids of the HIV-1 structural Gag protein was added to I3-01. To promote ESCRT recruitment, the 52-residue HIV-1 Gag p6 peptide (p6Gag) was added to the C terminus. EPN-01 could be detected in the culture supernatant, whereas no peptides without membrane-binding element were released, demonstrating its necessity of binding-membrane for the release of nanocages. In addition, a nanocage with a size of about 25 nm was observed owing to the presence of the designed self-assembly unit. Moreover, many different membrane-binding, self-assembly, and ESCRT-recruiting units were carried out to ensure the formation of nanocages, coated with host cell membrane and released from cells. A key feature of the proposed strategy is that it enables control over the biogenesis and contents of the materials through modification of EPN protein sequences. Enveloped nanocages are highly modular and tolerant to substantial alterations, demonstrating that they could be engineered to incorporate a series of properties and functions tailored to desired applications. Proteins which work within cells could be efficiently delivered across the cell membrane *via* the aforementioned strategy. In addition, the protein was packaged into nanoscaled vesicles to mimic the virus; the association of AAV with exosomes have been developed for gene therapy. Moreover, the biosynthesis strategy to construct biomimetic exosomes was utilized to improve the delivering efficiency.

### 2.2 Cargo Packaging

Until now, the drug loading approaches of exosomes and biomimetic exosomes have been divided into two main groups, the pre-loading methods and the post-loading methods.

In pre-loading approaches, the drug is first generated or loaded in the parental cells. Subsequently, these vesicles which were obtained by isolation or generation from cells have been preloaded into the desired drugs. By utilizing cell engineering techniques, the cells could be used to generate exosomes or biomimetic exosomes. It was a useful strategy for loading cargo including protein and nucleic acids into vesicles which could be produced in cells.

In post-loading approaches, the cargo was packaged into exosomes after isolation by different methods. Cargo packages with high yields have been developed by liposome engineering. A high ratio of cargo loading was successfully achieved in comparison to electroporation, which was utilized earlier. Generally, most drugs were loaded by passive loading with low loading rate. Active loading was an effective method to improve the encapsulation efficiency of drugs with amphiphilic properties. The loading approach of amphiphilic drugs such as DOX and camptothecin was passive, which included gradient ammonium sulfate and pH strategies. The cargo was loaded into vesicles derived from red blood cells through the active loading method, which was proposed by Zhang and his colleagues (Krishnan et al., 2021). To retain a fluorescent dye, the content of cholesterol in the membrane should be increased. The fluorescence intensity gradually increased with the increase in cholesterol content. When the percentage composition for cholesterol, the fluoresce signal reached its maximum. Using similar strategies, Vogelstein and coworkers have constructed an active method to load hydrophobic drugs, which was at a low drug loading rate *via* passive loading (Sur et al., 2014; Wang et al., 2022). Hydrophobic drugs were first packaged into modified β-cyclodextrins with weak basic groups, which facilitate to load drugs. By utilizing pH gradients, these cyclodextrins could subsequently carry drugs to pass through the lipid bilayer of liposomes. These strategies could be expanded to the package drugs into biomimetic exosomes.

### 2.3 Advantage and Disadvantage of Synthetic Strategies for Biomimetic Exosomes

Although the techniques to generate exosomes have made great progress, several drawbacks such as scalable production, isolation, purification, and cargo loading should be circumvented for their clinical application. In view of this, artificial exosomes have been carried out through bio-nanotechnology, particularly the development of fully synthetic exosomes. Currently, some problems remain to fabricate biomimetic exosomes, which include low output, time-consumption, and characterization (Table 1).

**TABLE 1:** Advantages and disadvantage of synthetic strategies for biomimetic exosomes.

	Method	Yield	Cargo loading	Stability	Reference
Top-down	Extrusion	High	Lack selectivity	Good	Jang et al. (2014)
	Sonication	High	Lack selectivity	Good	Go et al. (2019)
	Nitrogen cavitation	High	Lack selectivity	Good	Gao et al. (2016)
	Cellular blebbing	High	Lack selectivity	Good	Ingato et al. (2018)
Bottom-up	Liposome-based	High	Selectivity	Poor	Kim et al. (2018)
	Protein-based	High	Selectivity	Good	Dautel and Champion (2021)
	Fully synthetic exosomes	High	Selectivity	Good	Staufer et al. (2021)
Biological approaches	Derived from virus	Low	Selectivity	Good	Votteler et al. (2016)

Through top-down fabrication, cell membranes used as raw materials endowed the biomimetic exosomes with wide applicability because these vesicles showed a high similarity in physiochemical and biological characteristics to exosomes. Extrusion was the most widely applied strategy to produce a large number of exosome-mimetic vesicles. By applying this technology to cells, as much as 100-fold on the amount of biomimetic exosomes has been achieved in comparison to that of natural exosomes. Furthermore, conventional laboratory centrifuges were applied for the scale-up generation of biomimetic exosomes. However, ultracentrifugation was indispensable during isolation and purification after serial extrusion by using the top-down strategy. The fabrication of final vesicles was time-consuming. In addition, lack of selectivity in cargo sorting in the preparation of biomimetic exosomes was presented because the surrounding medium was packaged into biomimetic exosomes in the self-assembly of membrane debris. Nevertheless, the production sustainability was limited in the generation of biomimetic exosomes owing to cells as raw materials.

The bottom-up fabrication was capable of large-scale production of these vesicles with various forms, which was similar to the liposome generation. Since synthetic materials were easily available for large-scale processing, biomimetic exosomes could be obtained with a mass of vesicles. In order to mimic the function of natural exosomes, liposomes were modified with multiple components, leading to poor stability of liposomes and complex preparation processes. Even so, it was hard to mimic the component of natural exosomes. As a result, the function of exosomes was difficult to be completely reproduced by biomimetic exosomes on the basis of the bottom-up fabrication.

## 3 Application of Biomimetic Exosomes

In recent years, nano-delivery systems have received extensive attention due to their ability to deliver drugs to targeting lesions (Geranpayehvaghei et al., 2021). Therapeutic agents included small molecules and macromolecules such as proteins and nucleic acids. Nucleic acid drugs were composed of siRNA, shRNA, miRNA, antisense oligonucleotide (ASO), CRISPR/Cas 9 system, plasmid DNA, and mRNA. Similar to natural exosomes, biomimetic exosomes could transport these therapeutic agents into cells. Compared with other carriers, biomimetic exosomes showed good biocompatibility and possessed a targeting ability due to the presence of proteins or peptides on the surface of these nanovesicles. According to the types of molecules, the cargo delivered by biomimetic exosomes could be divided into four groups: small molecules, proteins, RNA, and DNA.

### 3.1 Small Molecule Delivery

**FIGURE 4:**
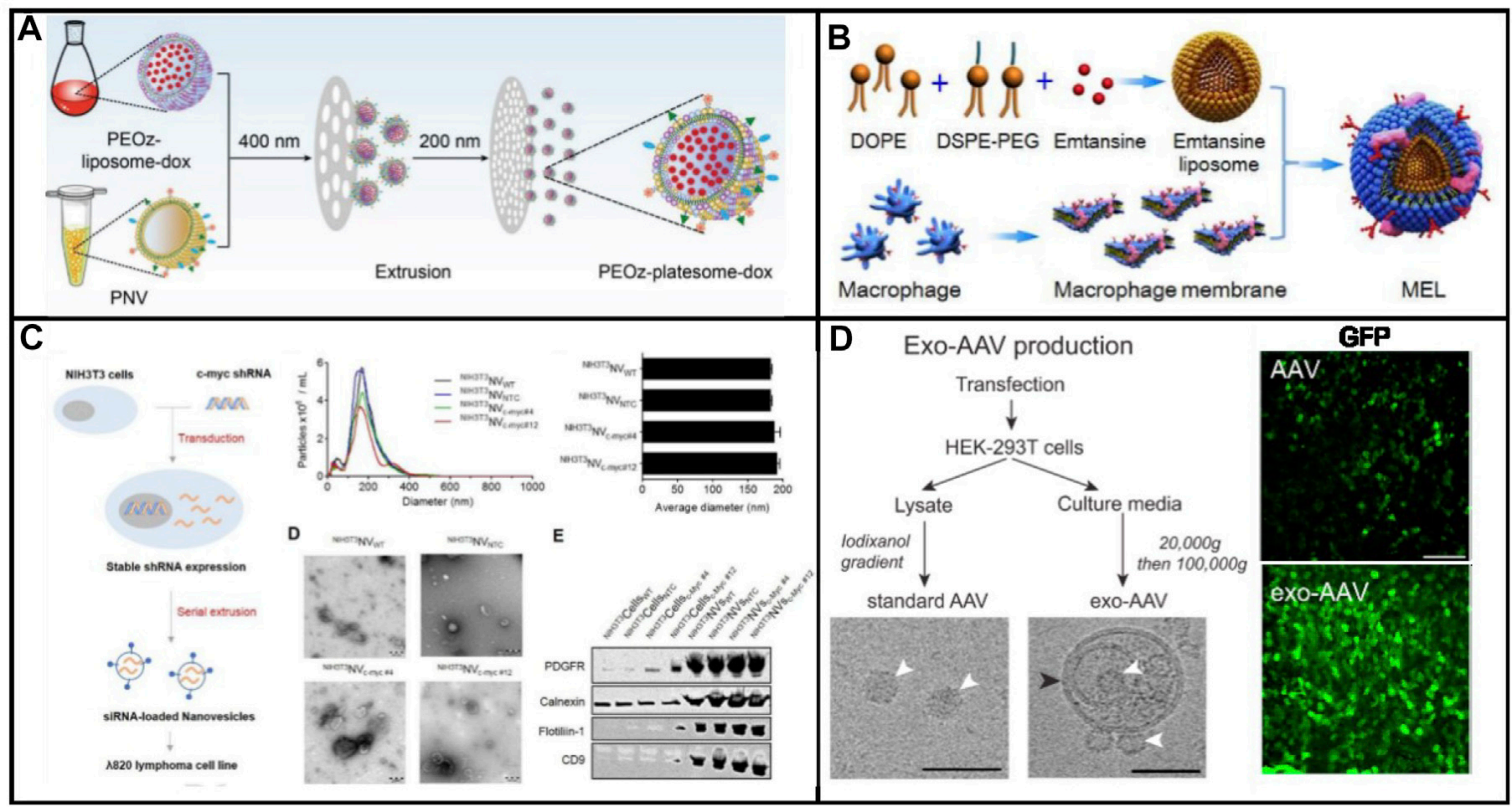
**(A)** Schematic illustration of biomimetic exosomes to deliver small molecule (Dox) (Liu et al., 2019). **(B)** Biomimetic exosomes to deliver protein (emtansine) for suppressing lung metastasis of breast cancer (Cao et al., 2016). **(C)** Scheme illustration of endogenous siRNA loading into nanovesicles (Lunavat et al., 2016). **(D)** Scheme of the preparation of standard (conventional) AAV and exo-AAV production workflow (Ramakrishnaiah et al., 2013).

Hydrophobic drugs could be loaded into phospholipid bilayers through hydrophobic interaction, while hydrophilic drugs were encapsulated into the core of artificial exosome. A pH responsive artificial exosome was constructed by Liu et al. (2019) *via* a convenient way for targeting delivery toward tumors. As can be seen in Figure 4A, platelet membranes and liposomes, which were functionalized with a pH responsive unit, were merged to construct the proposed nanocarrier. The platelet membranes of the vector endow the ability to escape from the immune system; pH responsive liposome endows the vehicle to release cargo selectively in the acidic microenvironment in lysosomal. Furthermore, the therapeutic effect toward tumors was better than that of plateletsome without a pH responsive unit or pH-sensitive liposome. Recently, biomimetic exosomes were designed for the delivery of various therapeutic drugs to meet treatment needs (Jang et al., 2013; Bozhao Li et al., 2021; Liao et al., 2021). The constructed nanocarriers were expected to treat a variety of diseases through chemotherapy, immune therapy, and phototherapy.

### 3.2 Protein Delivery

Compared with small drugs, protein drugs have the advantages of high activity, high specificity, and low toxicity. So far, more than 130 drugs have been approved by the FDA for the treatment of various diseases (Sanchez-Moreno et al., 2018). However, delivering proteins across cell membranes is a challenge, and as such, most protein drugs are developed with membranes as targets. Intracellular protein delivery is a huge obstacle to its development as a universal treatment. The progress of intracellular protein delivery will promote the development of protein drugs. The impermeability of cells is a major obstacle to the intracellular delivery of recombinant proteins. Endosomal escape is an effective way to overcome the cell barrier, but this strategy still presents a very big challenge. Therefore, there is an urgent need to develop a universal method for protein delivery. Natural exosomes themselves carry proteins, which are excellent carriers and deliver a variety of proteins. The biomimetic exosome delivery protein has the advantages of easy availability, easy functionalization, and large-scale production. Cao et al. (2016) developed a nanovesicle to deliver emtansine to metastatic sites for the treatment of cancer metastasis of breast cancer. As shown in Figure 4B, the nanomedicine was composed of macrophage membranes, liposomes, and emtansine. For the first time, a pH-responsive liposome was used to load emtansine, which was toxic to cancer cells. Subsequently, an isolated macrophage membrane was used to coat the liposome, ensuring the ability of nanomedicine toward metastases foci in the lung. The mixing between the liposome and macrophage membrane was conducted through extrusion. High expression of α4 and β1 integrins contributed to the enhancement of the cellular uptake of emtansine, where a 25% reduction in the cellular uptake of emtansine-loaded liposome is compared with that of macrophage membrane functionalized liposome. This was due to the interaction between α4β1 integrin and VCAM-1 in cancer cells that contributed to specific metastasis-targeting toward cancer metastasis. In particular, the specific metastasis targeting ability and anti-metastatic activity of MEL were evaluated in a lung metastatic breast cancer model.

### 3.3 RNA Delivery

Owing to its intrinsic capacity, various kinds of RNA species could be transferred by exosomes and other extracellular vesicles from one cell to another. As a consequence, these vesicles have been utilized as carriers for the delivery of therapeutic RNA molecules. Nevertheless, the rare production of exosomes limited its practical application field. Alternatively, biomimetic exosomes could serve as carriers to deliver RNA and DNA into cells. Lunavat et al. (2016) prepared artificial exosomes derived from cells *via* multiple extrusion, and thereafter these vesicles were loaded with a specific siRNA *via* electroporation (Figure 4C). These nanomedicines were taken up by cells effectively. The proteins in the generated nanovesicle were similar to those of the plasma membrane of parent cells. Subsequently, a decreased level of GFP was observed in the nanovesicle, which loaded GFP siRNA, indicating the reduction of target genes. In addition, the expression of C-myc was decreased after treatment with siRNA, which was loaded into biomimetic exosomes, demonstrating that these biomimetic exosomes could be served as nanomedicine to treat diseases which were caused by the overexpression of C-myc.

In general, biomimetic exosomes can efficiently protect their payload against degradation, although the active targeting efficiency is currently debated for both systems. Similar to exosomes, biomimetic exosomes can deliver small molecules and large biological molecules (RNA and proteins). Genetic materials are fragile in the human body, so they must be protected to take effect. mRNAs and viral or nonviral plasmid DNAs are the common genetic materials used in gene therapy. Encapsulation in nanostructures protects the AAV vectors from the host immune system and delivers them into cells across the plasma membrane. Recently, Ramakrishnaiah et al. (2013) reported that hepatocyte-derived exosomes containing hepatitis C virus (HCV) RNA can activate innate immune cells (Figure 4D). In the presence of neutralizing antibodies, the virus could be transferred by exosomes. Exosomes from HCV-infected cells were capable of transmitting infection to naive human hepatoma Huh7.5.1 cells and establishing a productive infection. Even with sub-genomic replicons, lacking structural virus proteins, exosome-mediated transmission of HCV RNA was observed. Treatment with patient-derived IgGs showed a variable degree of neutralization of exosome-mediated infection compared with free virus. Therefore, biomimetic exosomes can be used as vectors to deliver plasmids, mRNA, etc., for gene therapy. Furthermore, exosome-AAV hybrid biomimetic exosomes combined the advantages of viral vectors and nonviral vectors, which can escape from the immune system and significantly reduce the immunogenicity of the vector. The Maguire group has developed an artificial exosome as a vector to deliver genes toward all inner hair cells (Gyorgy et al., 2017). Compared with the adeno-associated virus, the GFP expression delivered by artificial exosomes was significantly higher than that of AAV. Exo-AAV shows no toxicity *in vivo* as assayed by tests of auditory and vestibular function. Finally, exo-AAV1 gene therapy partially rescues hearing in a mouse model of hereditary deafness.

### 3.4 DNA Delivery

Nucleic acid drugs contain siRNA, shRNA, miRNA, antisense oligonucleotide (ASO), CRISPR/Cas 9 system, plasmid DNA, and mRNA, which have captured much attention for therapeutic treatment of a variety of diseases and disorders. In order to prevent degradation *in vitro* and *in vivo*, different vehicles such as virus and non-virus have been developed to deliver these nucleic acids. In addition to the aforementioned delivery of RNA-based drugs, biomimetic exosomes have also been used to transfer DNA including plasmid and antisense oligonucletide to targeting cells. In 2021, the Lee group utilized biomimetic exosomes to efficiently deliver plasmid against glioblastoma with low toxicity (Han et al., 2021). The biomimetic exosomes consist of three units including cell membrane, PEI25k, and plasmid DNA. The cell membrane which was extracted from C6 rat glioblastoma cells could prolong circulation time by reducing the clearance of the reticuloendothelial system. Meanwhile, cell membranes in nanoparticles decrease their toxicity before reaching the target location *via* preventing drug leakage. PEI25k was a positively charged polymer, which could act as a vehicle by the formation of polymer/pDNA complex. The biomimetic exosomes were stable with a size of around 120 nm owing to the surface charge of approximately −32mV. Interestingly, no significant change was observed in the size of nanovesicles compared with nanoparticles without cell membranes, indicating a high stability of biomimetic exosomes. Furthermore, the biomimetic exosomes showed high transfection efficiency in a serum containing medium as compared to nanoparticles without cell membranes. After treatment with the prepared nanoparticle in glioblastoma-bearing mice, obvious reduction in tumor size was observed, demonstrating that biomimetic exosomes could be served as carriers for gene therapy.

Similar to the delivery of plasmid, ASO, which was a negatively charged DNA, could be transported by biomimetic exosomes to treat various diseases. Due to their strong anionic nature of ASOs, it was difficult to package the ASOs into nanovesicles. When using biomimetic exosomes for delivery of ASO, electroporation was a general approach to load these therapeutics into vesicles. Electroporation could increase the permeability of nanovesicles, therefore facilitating cargo into vesicles. In addition, electroporation could transfer more than two different ASOs into the same vesicles. Oieni et al. (2021) reported a scalable preparation strategy to generate gene vehicle for delivery of ASOs targeted to miRNA-221. Under low voltage conditions (700V), about 30% ASOs was encapsulated in biomimetic exosomes. To improve the targeting of biomimetic exosomes toward target tissue, cell membranes from human mesenchymal stem cells were employed to act as coating of nanoparticles. These nanovesicles could efficiently uptake by MSCs without endo-lysosomal degradation at 24 h after treatment with biomimetic exosomes. Subsequently, the miR-221 expression was dramatically decreased in MSCs on subjecting these nanomedicines. The proposed method could be extended to deliver different payloads toward various tissues with high specificity.

### 3.5 Smart Nanomedicine

**FIGURE 5:**
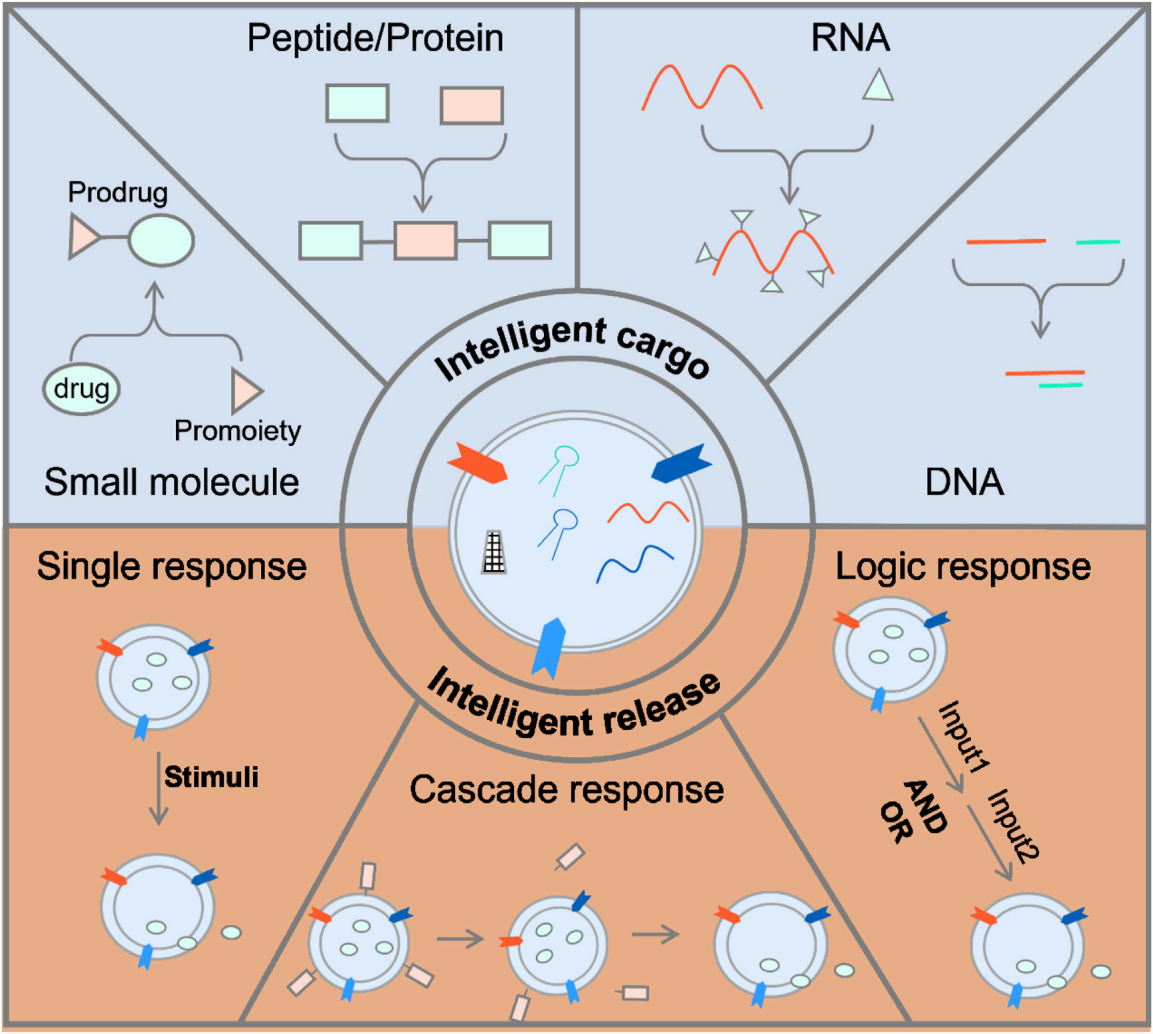
Strategies for smart nanomedicine based on biomimetic exosome.

However, due to the nonspecific release of the delivery system and rapid clearance in the blood, the therapeutic effect was limited, which required an increase in the dose of the drug and caused side effects (Meng et al., 2019). Nonspecific release referred that the drug is not delivered to the target location in advance before release (Tiwari et al., 2012). Another reason is that the drug is not released at the target location, so that it recirculates in the blood, and the drug is released in the process. Mu et al. (2020) Therefore, it is necessary to develop smart nanomedicine by using biomimetic exosomes so that it can only be released in a specific lesion site. As shown in Figure 4, there are two types of smart nanomedicine based on biomimetic exosomes: the smart drug and the smart drug delivery system. As shown in Figure 5, there are two types of smart nanomedicines based on biomimetic exosomes: the smart drug and the smart drug delivery system.

#### 3.5.1 Smart Drugs

Intelligence drug is a drug, which is inactive during the delivery process, and decomposes to release the original drug after reaching the target location to exert its therapeutic effect. The aforementioned strategies could improve the metabolic stability of the drug (Rautio et al., 2008; Walther et al., 2017; Rautio et al., 2018). Drugs have been classified into two types: small molecules and macromolecules (peptide, proteins, antibodies, and nucleic acids). Small molecule drugs could enhance their metabolic stability and reduce the toxicity caused by off-target *via* modifying functional groups (Rautio et al., 2008). Peptides were amino acids linked through peptide bonds. Biomacromolecule drugs could be incorporated with caging groups. The initial functions of biomacromolecules were restored upon removal of these caging groups (Diemer et al., 2020). Peptides could also constitute a variety of biological macromolecule-proteins that are essential for cell life activities (Lian and Ji, 2020). Peptides can increase their accumulation in specific tissues by adding targeting groups, adding transmembrane peptides to improve membrane penetration efficiency and endosome escape efficiency, adding self-assembly modules to increase drug stability, increasing response groups, and promoting drug release at the targeted location (Newman and Benoit, 2018). Stimulus-responsive groups can be roughly classified into enzyme-responsive (He et al., 2016), pH-responsive (Dharmayanti et al., 2021), and redox-responsive. By taking advantage of biotransformations, nucleic acids which were modified with specific groups were capable of transferring to bioactive nucleic acids in living cells.

#### 3.5.2 Smart Release

The smart drug carrier with a unique stimulate-response mechanism can make response adjustments to changes in related physical and chemical properties under the stimulation of exogenous or endogenous factors, control the release of the drug to the target site, and automatically adjust the rate of drug release Li et al. (2019). Smart deliveries have shown great promise in the field of material science and pharmacy (Huang et al., 2019). Due to the advantages of improving the bioavailability of drugs in the body and reducing their toxic and side effects, intelligent drug carriers will play an increasingly important role in future clinical treatments (Pan et al., 2021). The main potential mechanism of nanomedicine design is the enhanced permeability and retention effect (EPR) (Duan et al., 2021). Nevertheless, a number of recent studies have shown that less accumulation of nanomedicine was observed on account of poor therapeutic effect. Therefore, researchers have tried to improve the EPR effect or reduce the characteristics of the anti-EPR effect and found that strategies to improve the delivery of multifunctional nano-drugs such as regulating the abnormal tumor microenvironment and stimulus-responsive nano-drugs can enhance the anti-cancer drug delivery system.

## 4 Challenges and Perspective

On account of multiple drawbacks including low stability and unsatisfactory pharmacokinetic behavior, the naked therapeutic agents showed limited curative effect during clinical application. To address this issue, a series of delivery systems have been constructed to improve the therapeutic effect and reduce side effects over the past decades. Biomimetic exosomes are composed of reasonably designed lipids, proteins, and RNA, which have similar structures to liposome or exosome. Considering the disadvantages of lipid nanoparticles and liposomes, biomimetic exosomes may be the basis of personalized nanomedicine in terms of drug delivery system in the future, which is characterized by a wide range of sources, low price, stable physical and chemical properties, and good biocompatibility. In comparison with natural exosomes, biomimetic exosomes are much easier to be synthesized on a large scale. However, clinical trials of biomimetic exosomes have just begun, and biomimetic materials produced through synthetic strategies have not yet been used for clinical transformation. The main challenge is that there are no standard methods in preparation, characterization, and biocompatibility. In addition, a fully automated purification method is required for the preparation of biomimetic exosomes. To achieve large-scale production, the bottom-up method to produce biomimetic exosomes can be synthesized by a method similar to the synthesis of liposome-microfluidics. The fully synthesized biomimetic exosomes are easier to modify, and the modification of antibodies and other functional groups can help to control the release of biomimetic exosomes under a controllable spatiotemporal manner. In the future, new and multifunctional biomimetic exosomes will be developed through biotechnology, nanotechnology, and chemical technology as personalized nanomedicine.
